# *Clostridium difficile* Infection and Inflammatory Bowel Disease: A Review

**DOI:** 10.1155/2011/136064

**Published:** 2011-09-12

**Authors:** Preetika Sinh, Terrence A. Barrett, Laura Yun

**Affiliations:** Division of Gastroenterology, Feinberg School of Medicine, Northwestern University, 676 N St Clair Street, Suite 1400, Chicago, IL 60611, USA

## Abstract

The incidence of *Clostridium difficile* infection (CDI) has significantly increased in the last decade in the United States adding to the health care burden of the country. Patients with inflammatory bowel disease (IBD) have a higher prevalence of CDI and worse outcomes. In the past, the traditional risk factors for CDI were exposure to antibiotics and hospitalizations in elderly people. Today, it is not uncommon to diagnose CDI in a pregnant women or young adult who has no risk factors. *C. difficile* can be detected at the initial presentation of IBD, during a relapse or in asymptomatic carriers. It is important to keep a high index of suspicion for CDI in IBD patients and initiate prompt treatment to minimize complications. We summarize here the changing epidemiology, pathogenesis, risk factors, clinical features, and treatment of CDI in IBD.

## 1. Introduction

*C. difficile *is an anaerobic gram-positive spore forming bacilli causing infectious colitis traditionally in the elderly, hospitalized patients, or those with a history of antibiotic exposure [1, 2]. The trend has changed with increasing numbers of younger, post transplant, and immunocompromised patients acquiring CDI [3, 4]. Patients with inflammatory bowel diseases (IBD), ulcerative colitis (UC), and Crohn's disease (CD) are also acquiring CDI in increasing numbers and have worse outcomes with higher rates of hospitalization, surgery, and mortality as compared to non-IBD CDI patients [5–7]. The recognition of a new hypervirulent strain of *Clostridium difficile *called NAPI/B1/027 has been linked to the increase in health care burden in the last 10 years. The annual health care cost of *C. difficile* infection (CDI) in the United States is between $436 million to $3 billion according to published data in the last decade [8–11]. 

## 2. Epidemiology

The role of *C. difficile *in antibiotic associated diarrhea and pseudomembranous colitis was first described in the late 1970's [12, 13]. A number of studies have reported a higher prevalence of CDI in IBD patients [5–7, 14, 15]. Cases of *C. difficile* among UC patients reported in a nation wide data analysis by Nguyen et al. between 1998 and 2004 were 3.73% as compared to 1.09% for Crohn's disease and 0.45% for general inpatient admissions. They also found that the incidence of CDI in UC patients had doubled from 2.66% to 5.12% over those 7 years [14]. A study based on a larger cohort of IBD patients in the United States reported similar results and found that CDI was more common in UC patients (2.8%) as compared to the general inpatient population (0.4%) [15]. There was no significant increase in the overall prevalence of CDI in all Crohn's disease patients over the study period (1993–2003), but there was an increase in cases of CDI in CD patients with large bowel disease from 1.22% to 2.31% [15]. Rodemann et al. around the same time period (1998–2004) reported adjusted odds ratio of all IBD, UC, and CD admission with CDI as 2.9 (95% CI, 2.1–4.1), 4.0 (95% CI, 2.4–6.6), and 2.1 (95% CI, 1.3–3.4), respectively with doubling of CDI admissions in patients with CD and tripling in those with UC [7]. More recent data from a retrospective observational study by Issa et al. found that the rate of CDI in IBD patients increased from 1.8% in 2004 to 4.6% in 2005. The majority of cases reported in 2005 were colonic IBD (91%) and outpatient acquired infections (76%) [6]. It was argued by Powell et al. that the relative increase in CDI in UC compared to CD was due to the extent of colonic involvement in UC rather than the difference in nature of the two diseases [16]. Their preliminary data suggests a much higher incidence of CDI in left sided and extensive disease as compared to distal disease. Hence, the incidence of CDI has not only increased in the general population but also to a greater extent in IBD patients.

## 3. Pathogenicity of *C. difficile*

*C. difficile *spores are transmitted by fecal-oral route, which when ingested find an adequate environment and pH in the bile of the small bowel to germinate into their vegetative forms and subsequently colonize the intestine. Toxin-induced damage of the mucosal barrier is the main pathogenic mechanism of *C. difficile. *Two main types of toxins, namely, toxin A and toxin B are important for pathogenesis. Both have the ability to be enterotoxic and cytotoxic though traditionally toxin A is considered enterotoxic which causes disruption of the intestinal epithelial lining giving way to cytotoxic toxin B [17]. This leads to activation of a cascade of proinflammatory cytokines and leukotrienes like tumor necrosis factor (TNF), interleukin (IL)-6, IL-8, IL-1*β*, leukotrienes B4, and interferon-*γ* [18, 19]. These cytokines account for enhanced permeability, diarrhea, epithelial apoptosis, and ulceration.

Genes encoding toxin A (*tcdA*) and toxin B (*tcdB*) can be upregulated (*tcdR) *or downregulated (*tcdC)* by genes in the same loci. The local effect of these toxins is mediated by internalization of toxin via an endosome in epithelial cells leading to a sequence of conformational changes that release the TcdB toxins catalytic-DXD glycosyltransferase domain. Subsequent glycosylation of the target RhoGTPase disrupts the cellular cytoskeleton and causes cell death [20–22]. 

Most strains of *C. difficile* produce both toxin A and B. The proinflammatory mediators induced by these toxins are responsible for the formation of pseudomembrane. It is interesting to note that the classical pseudomembrane is not a frequent finding in IBD-associated CDI [6]. One possible explanation is that the weakened intestinal lymphoepithelial environment of a chronically active IBD patient is unable to mount an adequate inflammatory response to form a pseudomembrane. Immunomodulating drugs may also contribute by altering mucosal inflammatory responses. A third toxin called binary toxin is produced by some strains though the exact role is not well understood [23].

## 4. Hypervirulent NAP1/B1/027 Strain

The emergence of a hypervirulent strain NAPI/B1/027 at the beginning of the last decade coincided with the increase in CDI cases [24]. In early 2000, an atypical strain that was group B1 (restriction-endonuclease analysis REA), type NAP1 (North American PFGE type1) and ribotype 027 (polymerase chain reaction PCR) was isolated from outbreaks and found to exhibit hypervirulent features that caused more severe disease [25]. 

The atypical NAP1/B1/027 strain has several features that contribute to its clinical presentation. It is resistant to fluoroquinolones. It has mutated *tcdC* that negatively regulates *tcdA *and *tcdB. *It produces 16 and 23 times more TcdA and TcdB as compared to the common toxinotype 0 strains in vivo. It produces a third toxin called binary toxin whose function in CDI is unclear. This strain is a toxinotype III as compared to most other strains that are toxinotype 0 [26]. Toxinotyping is based on pathogenic gene loci in bacteria that carry genes encoding the toxins.

## 5. Risk Factors

Risk factors associated with CDI in the general population are antibiotic use, older age, residence in long-term care facilities, hospitalization, immunosuppression, greater comorbid burden, cancer, gastrointestinal disorders, and gastrointestinal surgery (Table 1). In 2005, the Centers for Disease Control (CDC) reported cases of fatal CDI in young and peripartum patients who had no prior exposure to antibiotics or medical facilities. These patient groups were not thought to be the typical at-risk population [4]. 

Risk factors in IBD patients include older age, antibiotic use, steroids, greater comorbid burden, and those with colonic disease. Increasing age in IBD patients has been shown to be a risk factor for CDI [9, 27, 28], although IBD patients with CDI tend to be younger than those with CDI in the general population. Antibiotic use alters the normal bacterial flora and can lead to CDI [29–31]. Broad-spectrum antibiotics are more likely to cause CDI primarily because of a greater disruption of the normal microflora. Nearly all antibiotics have been reported to cause CDI even those with therapeutic properties (e.g., metronidazole and vancomycin) [32]. Clindamycin was one of the initial culprits, but more recent outbreaks of CDI tend to be associated with broad-spectrum antibiotics like fluoroquinolones. These case reports emerged with the reporting of the NAP1/B1/027 strain that is resistant to this drug [33, 34]. Antibiotic exposure is an important factor altering host microbiota and most of the studies show an exposure rate close to 60% in IBD patients with CDI [6, 35]. Even though antibiotic exposure is an important risk factor for CDI in IBD, the absence of it should not lower the suspicion of CDI.

Antibiotics are not the only drugs that predispose to CDI. Immunosuppression is a risk factor for CDI. Immunosuppression with corticosteroids has been associated with CDI in IBD patients. A study of IBD patients in British Columbia between 2001 and 2006 showed a threefold increase in risk of CDI with corticosteroid use, with or without immunomodulators therapy (RR 3.3 95% CI 1.88–6.10) [36]. It is not clear if the immunomodulating drugs like azathioprine, 6-mercaptopurine, and methotrexate enhance the risk of CDI. Issa et al. reported in their single tertiary center study an odds ratio of 2.56 (*P *= 0.011 95% CI 1.28–5.12) for CDI for patients on maintenance immunosuppression. There was a greater risk with purine analogs (i.e., azathioprine or 6-mercaptopurine) with or without infliximab [6]. However, a recent observational study of CDI in IBD patients found no association between immunomodulators and the risk of CDI, emphasizing the need for more research in this area [37]. Thus far, there is no evidence from these retrospective studies that biological agents like infliximab are associated with an increase risk of CDI. In addition, those IBD patients with greater comorbid burden are at increased risk for CDI. Nguyen et al. found a 13% increase in risk of CDI with each 1-point increase in the Charlson's comorbidity burden index [14]. Studies examining the association of proton pump inhibitor (PPI) use and CDI in the general population have shown conflicting results and more data is needed with regards to the IBD population [33, 38–43]. Larger prospective studies will be needed to better define this relationship.

## 6. Presentation of *C. difficile* Infection in IBD

### 6.1. Clinical Features and Outcome

An episode of CDI is characterized by increase in stool frequency, diarrhea, fever, nausea, abdominal pain, and tenderness with nonbloody stools. The presentation can range from asymptomatic carriage to severe infection with toxic megacolon and ileus requiring surgical intervention [44]. *C. difficile* in IBD may show atypical features like frequent bloody stools and presentation in younger patients with no prior exposure to hospital facility [6, 45]. It is important to keep a high suspicion for CDI in these patients. Even in the absence of diarrhea if constitutional symptoms and laboratory findings like leukocytosis with left shift are indicative of a possible infectious source, it is prudent to rule out *C. difficile.* Articles highlighting the role of CDI in IBD flares were published as early as the 1980's [46–49], but there was no clear consensus on checking *C. difficile* toxin in IBD flare [46–52] until recent data by Meyer et al. and others emphasized the importance of *C. difficile* during IBD relapse [5–7, 53–55]. 

CDI worsens IBD outcome. IBD patients with CDI have higher rates of inpatient endoscopy and surgical procedures, longer length of hospital stay, and higher mortality as compared to non-CDI IBD or CDI-only patients [5, 6]. In a single center study at the University of Wisconsin in 2004-2005, more than half of the infected IBD patients required hospitalization and 20% required colectomy [6]. Another single center study analyzed the outcome of UC patients with and without CDI over a period of one year. The investigators found significantly more hospitalizations (58% versus 27%) and higher rates of colectomy (44.6% versus 25%) in *C. difficile* positive- as compared to *C. difficile *negative-UC patients in the follow-up year. Differences in the colectomy rates and the lengths of hospital stay at initial admission were not statistically significant [55]. However, data analyzed by Anathakrishnan et al. from a nationwide inpatient sample (2003) showed that hospital stays were three-day longer in *C. difficile *positive subjects. The CDI and IBD group was six times more likely to undergo bowel surgery than the *C. difficile-*only group and had four times higher mortality than patients admitted to the hospital with IBD alone or *C. difficile* alone. The endoscopy and surgical rates in UC were higher than CD [5]. A recent retrospective observational study by Kariv et al. of inpatient and outpatient IBD cases showed that CDI does not increase the risk of colectomy in UC patients at 3 month followup [37]. A negative relationship between colectomy and CDI was also observed by Nguyen et al. in their nationwide survey of inpatients from 1998 to 2004 [14]. The improved outcome could be attributed to prompt pathogen-directed treatment for *C. difficile* infection in UC subjects [37]. This result contradicts previous data that showed higher colectomy rates in IBD patient with CDI than those without [5, 6]. Prospective studies need to be conducted to better address this question. 

Not only does CDI add to the morbidity and health care cost due to longer hospital stays and higher number of procedures, it also contributes to a higher case fatality rate in IBD patients (5.7%–18% in IBD and CDI versus. 1.4%–2.1% in IBD alone) [15]. The effect of CDI on CD is less pronounced as compared to UC. This may be due to less colonic Crohn's in the population studied and possibly higher use of metronidazole in CD [15, 56]. Even in studies that showed no increase in colectomy rates in *C. difficile* cases the mortality was higher as compared to non-IBD cases [14] arguing that there are other variables at play. Stratification of study patients based on disease severity and use of immunomodulators may help find answers to these questions. 

When comparing data, it is prudent to keep in mind the variability of testing for *C. difficile *colitis in various centers. Nevertheless, it is clear that CDI is detrimental to the clinical outcome of an IBD patient, already complicated by reduced host immunity from drugs, malnutrition, and physical stress (from surgery, infection, bleeding, etc.).

Small bowel CDI has been reported only in postcolectomy IBD patients. High ileostomy output accompanied by abdominal tenderness, nausea, fever, and high leukocyte count are the usual presenting complaints [57]. There have also been case reports of *C. difficile *causing diversion colitis and pouchitis in postcolectomy cases [58–61].

### 6.2. Endoscopic Findings

The endoscopic finding of pseudomembranes found in 50% of *C. difficile* infected patients (according to data from last decade), is less common (13%) [62] in IBD patients with CDI [6, 63, 64]. Pseudomembrane formation occurs when there is disruption of cellular cytoskeleton by toxins leading to ulcer formation. These ulcers leak out serum proteins, inflammatory cells, and mucus forming plaques on the colonic mucosa that cannot be removed by lavage [3]. Although IBD patients with pseudomembranes present more commonly with fever their clinical outcome is similar to patients without pseudomembranes, [62]. Other endoscopic features of CDI in IBD patients are nonspecific findings of edema, erythema, friability, and inflammation of the colonic mucosa [6].

## 7. Diagnosis

Anaerobic stool culture is the most sensitive test for diagnosing *C. difficile,* but it is labor intensive and has a turn around time of 48 hours. The cytotoxin assay test, which has a sensitivity of 67–100%, also requires 24–48 hours [65]. A combination of stool culture followed by identification of toxigenic culture is recommended as the standard for clinical tests [66].

On the other hand, enzyme immunoassay (EIA) for toxins A and B is rapid and more commonly used. The sensitivity of single sample testing is low (72%) [6, 67] and may increase by 10% with second stool testing (84%) [68]. Peterson et al. reported a sensitivity of 86% with three sample testing [67]. Although three sample testing increases the yield of EIA for *C. difficile* toxin detection it adds to the health care cost of the disease. Infectious Diseases Society of America (IDSA) does not recommend repeat testing during the same episode [66]. Binion et al. at the Medical College of Wisconsin found that in IBD patients the sensitivity is even less impressive (54%). With a second, third, and fourth specimen, it increases to 75%, 78%, and 92%, respectively [6]. The specificity and negative predictive values of this test are 98% each [67]. More data is awaited on newer testing modalities like real-time PCR that are rapid and sensitive. PCR has sensitivity and specificity of 86–100% and 93–98%, respectively [67]. The IDSA does acknowledge that there is no testing strategy that is optimally sensitive and specific. An interim recommendation made in the 2010 guidelines suggests using a two-step method that uses EIA detection of glutamate dehyrogenase (GDH) as the initial screening test followed by cellcytotoxicity assay or toxigenic culture for confirmation. At our institution all stool samples are screened with EIA for GDH. Toxin assay (EIA) is run on GDH-positive specimens. GDH-positive, toxin-negative samples are cultured for *C. difficile* isolates. Recovered isolated are cultured in broth to test for the production of toxin. More research needs to be done to determine the optimal diagnostic test for CDI in the general population as well as in IBD patients. 

Endoscopy is not considered diagnostic for CDI. Nonspecific findings on endoscopy are common in IBD-related CDI, but stool samples sent during colonoscopy can be useful. 

It is important for the physician to keep a high index of suspicion for CDI in IBD patients. Stool tests should also be performed in cases of IBD relapse, especially with a history of antibiotic use in the last couple of months [54].

## 8. Treatment

It is important to keep a high index of suspicion followed by prompt diagnosis and early treatment of *C*. *difficile *in IBD patients. In IBD local host defenses could be compromised due to altered gut microflora from the disease or concurrent use of immunomodulators making management challenging. Secondly, the symptoms and endoscopic appearance of active CD or UC are very similar to CDI. Thus, CDI poses a diagnostic and therapeutic challenge to the physician. Immunomodulators (including corticosteroids) treat IBD flares but could be detrimental for CDI [45] (Table 2). 

### 8.1. Antibiotics

**Figure 1:**
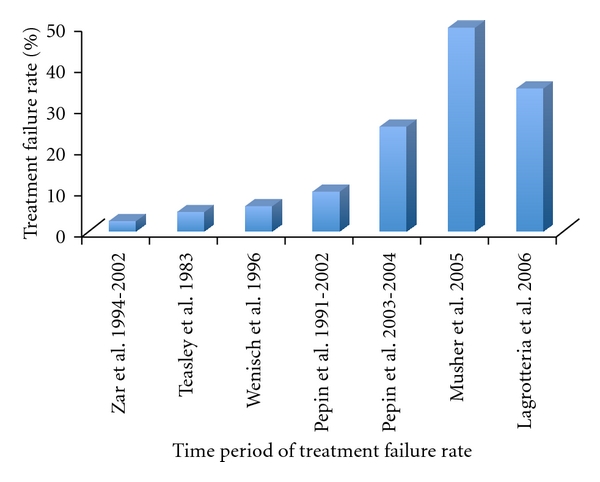
Treatment failure rates (%) of metronidazole in *C. difficle* infection: The rate of treatment failure with metronidazole has increased over the last 2 decades. Metronidazole treatment failure rates (%) however have increased the most after 2000 with the emergence of hypervirulent NAP1/B1/027 strain [66, 67, 69, 70, 78].

Metronidazole is the first line of treatment for mild-to-moderate CDI even though it is not FDA approved for this indication. The typical dose for mild-to-moderate CDI is 500 mg orally three times a day for 10−14 days. Metronidazole 500 mg intravenous four times a day is equally efficacious. For mild-to-moderate disease there is no evidence suggesting that it is less effective than vancomycin [69]. It is not only cheaper than vancomycin but also achieves effective concentrations in the colon by both oral and intravenous administration. The response rates range from 95% [70] to 50% [71] over the last 2 decades in all CDI cases. There is a watershed area that coincides with the emergence of NAP1/B1/027 strain after which there is increase in both initial treatment failure rate and recurrence rate [2, 24, 70–74] (Figure 1). Even though *C. difficile* resistance to metronidazole had been generally uncommon, recent reports indicate increasing resistance rate from 7.7% in 1994, 6.3% in 2002 to 12% in 2008 [75–77].

Oral vancomycin is the only FDA approved drug for CDI. The treatment failure rate of vancomycin is much less than metronidazole and is unaffected by the emergence of the new 027 strain (3.4%) [71, 72]. Vancomycin is indicated for treatment of an initial episode of severe CDI, second *C. difficile* recurrence, inability to tolerate metronidazole, and in pregnant females. In a randomized clinical trial by Zar et al., they found that the initial response rate with metronidazole (250 mg four times a day) and vancomycin (125 mg four times a day) was similar in those with mild disease (90% and 98%, resp., *P* = 0.36). However, in those with severe disease metronidazole had only a 76% cure rate compared to a 97% cure rate with vancomycin (*P* = 0.02) [72]. 

The optimal treatment regimen for CDI in IBD patients is not known. Failure rates of up to 50% have been reported in IBD patients with metronidazole [6, 79]. Given the worse outcomes in IBD patients with CDI, some institutions are practicing a more aggressive approach by using vancomycin as a first-line drug. By switching the regimen and rapid decrease in steroid dosing Issa et al. were able to reduce their institutions colectomy rate within a year [14, 36, 37]. By following a similar approach, Kariv et al. found that colectomy rates were lower in IBD patients with CDI compared to IBD patients without CDI. This effect was probably due to targeted antimicrobial treatment [6, 37]. Certainly for those patients with a high pretest probability (prior CDI, recent antibiotic exposure, etc.), empiric treatment with vancomycin should be considered while awaiting definitive diagnosis.

Studies that looked at other antibiotics like rifampin, bacitracin, rifaximin, nitazoxanide, and fusidic acid did not show superior efficacy compared to metronidazole or vancomycin in treating the first episode of CDI [69]. Fidaxomicin, a macrocyclic antibiotic, which is approximately eight times more active in vitro against clinical isolates of *C. difficile* than vancomycin was as effective as vancomycin in rates of clinical cure in a noninferiority trial (88.2% with fidaxomicin and 85.8% with vancomycin). This trial did not include patients with IBD but it would be interesting to evaluate these new drugs in this cohort [80]. A drug, teicoplanin, which is not available in United States has been shown to be slightly more efficacious than vancomycin with RR 1.21 (95% CI 1.00–1.46 *P* = 0.006), RR 1.82 (95% CI 1.19–2.78 *P* = 0.0006), and RR 1.43 (95% CI 1.14–1.81 *P* = 0.002) for initial symptom response, bacteriological cure, and initial bacterial resolution respectively [69]. 

Tigecycline, the first of a new class of broad spectrum antibiotics, the glycyclines, binds to 30S ribosomal subunit, inhibiting protein synthesis in a fashion similar to aminoglycosides, macrolides, and linezolid [81]. It has been approved for parenteral treatment of complicated intra-abdominal and skin infections. The use of tigecycline in treatment of refractory *C. difficile *infection has shown promising results [82, 83]. Tigecycline has higher mean fecal concentration (mean 5.6 mg/L, range 3.0–14.1 mg/L) after intravenous administration of 100 mg loading dose followed by 50 mg twice daily as compared to intravenous metronidazole (mean 0 mg/L, range 0–10.2 mg/L) [81, 84–86]. In critically ill patients, where there is doubt about adequate drug delivery of vancomycin through oral or enema route, intravenous tigecycline provides a reliable source of drug delivery and efficacy. Even though the results of tigecycline are promising it should be used with caution in severely ill patients because of risk of superimposed infection and other complications [87].

### 8.2. Immunomodulators

CDI can precipitate an IBD flare and optimal antibiotic therapy may not lead to symptom resolution. In patients who are not already on immunomodulators these drugs may need to be added to treat the underlying IBD. The optimal therapy is unclear and there is little data to guide us on the most appropriate strategy in this patient population. A recent retrospective multicenter European study compared 155 hospitalized IBD patients with CDI who were treated with antibiotics and immunomodulators or antibiotics alone. The primary outcome of death or colectomy within 3 months of admission was reported in 12% of patients treated with antibiotics and immunomodulators as compared to none in the group treated with antibiotics alone [45]. Immunomodulators included in this study were corticosteroids (more than or equal to 20 mg per day), thiopurines, methotrexate, cyclosporine, tacrolimus, or biological agent of any kind [45]. They found that use of 2 or more immunomodulators further increased the risk of complications (odds ratio 17, 95%; CI 3.2–91) [45]. Cyclosporine use did not reach statistical significance for the primary outcome. With respect to infliximab a recent study looking at the risk of CDI with infliximab in IBD patients did not show an increased risk [36]. Even though previously there was compelling evidence that immunomodulators increase the risk of CDI in IBD patients recent studies have raised doubts regarding this association.

### 8.3. Probiotics

Probiotics help restore the normal microflora of the gut. There have been some promising results with the use of probiotics in preventing antibiotic associated diarrhea or CDI [88, 89]. The best data exists for *Saccharomyces boulardi *which has been shown to be effective in preventing recurrent CDI [90]. There are studies that evaluate the primary and secondary preventive roles of probiotics but none with a focus on IBD patients. As of now there is insufficient data to recommend use of probiotics with antibiotics for primary prevention of CDI [66]. There is no data on use of probiotics alone for treating infection [91].

### 8.4. Intravenous (IV) Immunoglobulin

Host immunity is an important factor in CDI and elevated levels of immunoglobulins against toxin A and B are found in asymptomatic carriers of *C. difficile* [92]. IV immunoglobulins have been tried in about a dozen nonrandomized trials for treatment of refractory and severe cases [93–96]. There were promising case report data for severe infection but case control trials showed equivocal benefit and higher mortality [96–98]. At this time we recommend use of IV immunoglobulins (IVIG) only in the context of a prospective trial.

### 8.5. Surgery

Surgery is indicated in patients with CDI who have toxic megacolon, perforation hemorrhagic fulminant infection, and those refractory to medical therapy [37, 99]. Fulminant colitis occurs in 3–8% of *C. difficile *cases and the postoperative mortality can be as high as 50% [100]. Patients with history of IBD, recent surgery, and prior treatment with IVIG are at increased risk of developing fulminant colitis and early surgical intervention is key to avoiding refractory colitis and improving outcome [101–103]. Factors contributing to higher mortality in fulminant colitis, namely, longer hospital stay (more than 6 days), end organ failure, leukocytosis (>16,000/*μ*L), vasopressor requirement, and increased lactate, should guide early surgical intervention [103]. Subtotal colectomy with end ileostomy is the treatment of choice in CDI [86]. To improve the postoperative survival it is imperative to have a high clinical suspicion and focus on early intervention and careful patient selection for colectomy [100, 103]. 

### 8.6. Recurrent CDI

In general, *C. difficile * recurrence can occur in 15–20% of cases after the first episode and subsequent rates of recurrence are even higher (33–60%) [27, 44, 104]. Relapse is defined as infection within 7–14 days of treatment. The persistence of spores in the colon is a potential source of recurrent infections. There is limited data regarding risk of CDI recurrence in IBD patients. 

IDSA guidelines recommend treatment of the first episode of recurrent infection with a repeat dose of the first drug for 10 days. Patients with a second recurrence should be treated with vancomycin 125 mg four times a day for 10–14 days followed by a tapering regimen (125 mg 2 times a day for 1 week, 125 mg once a day for 1 week, then 125 mg every 2-3 days for 2–8 weeks) [70, 105, 106]. Fecal transplant for floral reconstitution has been tried in some refractory cases with success [107, 108]. Rifaximin has also been used in conjunction with vancomycin or alone over a tapering course with positive results and has been effective in IBD patients [109]. Probiotics (*Saccharomyces boulardii) *are promising candidates in combination with other antibiotics to prevent CDI recurrence in the general population [110]. Monoclonal antibodies against toxin A and B have been recently used with antibiotics to treat recurrent infection with some success in the general population [111]. The optimal treatment of recurrent disease in IBD patients is unclear and at this time should be treated like the non-IBD population until further studies are available.

## 9. Prevention

Environmental disinfection is foremost in the prevention of CDI. Contamination has been found on 49% of sites in rooms of *C. difficile *infected patients and 29% in rooms of asymptomatic carriers [112, 113]. Alcohol and ammonium-based cleaning agents act on the vegetative form and are not effective against spores; in fact, they encourage sporulation. Chlorine-based disinfectants and high concentration of vaporized hydrogen peroxide are sporicidal. Nosocomial transmission of *C. difficile* can be substantially decreased by adequate hand washing with soap and water defined as 15–30 seconds of hand washing and rinsing, followed by drying using a clean disposable paper towel [78, 113–121]. 1 : 10 dilution of bleach (concentrated sodium hypochlorite) is an effective cleaning solution [115, 122, 123]. 

Prophylaxis of patients on antibiotics or treating asymptomatic carriers is not useful in preventing spread of infection in the general population [114, 124–127]. Oral toxoid vaccines that were effective in cows and poultry were unsuccessful in human studies, likely due to the acidic environment of the stomach. Parenteral vaccines against *C. difficile* have been tried on small number of healthy volunteers and those with recurrent infection with IgG response over the threshold but larger studies that show IgA response are awaited [128–130]. 

## 10. Summary

Over the past decade CDI rates have accelerated in healthy and IBD patient populations. CDI carries special consideration for those with IBD. The risk of CDI is greater in IBD patients where it is linked to significant morbidity. CDI also increases the risk of IBD recurrence. CDI leads to worse outcomes in IBD patients and hence it is important to test IBD patients presenting with a flare and initiate therapy early. The subtype of IBD (UC > CD), host factors like immunosuppression, and extent of colonic involvement influence the outcome of the disease. With the emergence of a new hypervirulent strain NAP1/B1/027 in the last decade, the treatment response rate of metronidazole has decreased. Oral vancomycin is a good alternative to metronidazole in severe infection and in patients who cannot take metronidazole. There are no guidelines and limited research data available to address appropriate therapy in IBD patients and those on concurrent immunosuppressant therapy who acquire CDI. Prospective clinical trials are needed. In addition, new therapeutic modalities like immunoglobulins, fecal transplant are being explored for treatment of severe, refractory, and recurrent infection. A new drug, fidaxomicin, a macrocyclic RNA polymerase inhibitor was recently approved by the FDA for treatment of CDI [110, 131]. Overall, we recommend clinicians consider CDI with every flare of symptoms in IBD patients. Proper identification, treatment, and prevention of CDI in IBD patients greatly improve outcomes and preserve quality of life.

## Figures and Tables

**Table 1:** Risk factors for *Clostridium difficile *infection in IBD.

(1) Medications:
(a) Antibiotics
(i) More common
(1) Clindamycin
(2) Fluoroquinolones
(3) Broad spectrum penicillin
(4) Broad spectrum cephalosporins
(ii) Less common
(1) Metronidazole
(2) Vancomycin
(b) Corticosteroid use
(2) Disease related:
(a) Disease subtype: ulcerative colitis versus Crohn's disease
(b) Colonic involvement in IBD
(3) Hospitalization and exposure to hospital personnel
(4) Nonsummer months (20% higher rate)
(5) Advanced age
(6) Residence at long-term care facility

**Table 2:** Treatment of *Clostridium difficile *infection: IDSA guidelines: [66].

Disease category	Treatment
Initial episode, mild to moderate disease	Metronidazole 500 mg three times a day by mouth for 10–14 days

Initial episode, severe disease (uncomplicated)*	Vancomycin 125 mg four times a day by mouth for 10–14 days

Initial episode, severe infection (complicated)*	Vancomycin, 500 mg four times a day by mouth or by nasogastric tube, plus metronidazole, 500 mg every 8 hours intravenously. If complete Ileus, consider rectal instillation of vancomycin

For recurrence	Same as for initial episode

Second recurrence	Vancomycin in a tapered and/or pulsed regime


* Severe uncomplicated infection: white blood cell count of 15,000 cells/*μ*L or higher or serum creatinine level greater than or equal to 1.5 times the premorbid level. Severe complicated infection: hypotension or shock, ileus, megacolon (classification based on expert opinion by Infectious disease society of America (IDSA) [66].
